# Apparent diffusion coefficient values predict response to brachytherapy in bulky cervical cancer

**DOI:** 10.1186/s13014-024-02425-6

**Published:** 2024-03-13

**Authors:** Elizabeth E. Dong, Junqian Xu, Joo-Won Kim, Jason Bryan, Jewel Appleton, Daniel A. Hamstra, Michelle S. Ludwig, Alexander N. Hanania

**Affiliations:** 4 Department of Radiation Oncology, Dan L. Duncan Cancer Center, Baylor College of Medicine, Houston, TX USA; 2 Department of Radiology, Baylor College of Medicine, Houston, TX USA; 3 Department of Psychiatry, Baylor College of Medicine, Houston, TX USA; 5 Department of Radiology, Texas Children’s Hospital, 7200 Cambridge St, 77030 Houston, TX USA; 1 Smith Clinic Attwell Radiation Therapy Center, Harris Health System, Houston, TX USA

**Keywords:** Cervical cancer, Diffusion weighted imaging, Apparent diffusion coefficient, Interstitial brachytherapy

## Abstract

**Background:**

Diffusion-weighted magnetic resonance imaging (DWI) provides a measurement of tumor cellularity. We evaluated the potential of apparent diffusion coefficient (ADC) values obtained from post-external beam radiation therapy (EBRT) DWI and prior to brachytherapy (BT) to predict for complete metabolic response (CMR) in bulky cervical cancer.

**Methods:**

Clinical and DWI (b value = 500 s/mm^2^) data were obtained from patients undergoing interstitial BT with high-risk clinical target volumes (HR-CTVs) > 30 cc. Volumes were contoured on co-registered T2 weighted images and 90th percentile ADC values were calculated. Patients were stratified by CMR (defined by PET-CT at three months post-BT). Relation of CMR with 90th percentile ADC values and other clinical factors (International Federation of Gynecology and Obstetrics (FIGO) stage, histology, tumor and HR-CTV size, pre-treatment hemoglobin, and age) was assessed both in univariate and multivariate logistic regression analyses. Youden’s J statistic was used to identify a threshold value.

**Results:**

Among 45 patients, twenty-eight (62%) achieved a CMR. On univariate analysis for CMR, only 90th percentile ADC value was significant (*p* = 0.029) while other imaging and clinical factors were not. Borderline significant factors were HR-CTV size (*p =* 0.054) and number of chemotherapy cycles (*p =* 0.078). On multivariate analysis 90th percentile ADC (*p <* 0.0001) and HR-CTV size (*p <* 0.003) were highly significant. Patients with 90th percentile ADC values above 2.10 × 10^− 3^ mm^2^/s were 5.33 (95% CI, 1.35–24.4) times more likely to achieve CMR.

**Conclusions:**

Clinical DWI may serve to risk-stratify patients undergoing interstitial BT for bulky cervical cancer.

## Background

Cervical cancer is the fourth leading cause of cancer death among women globally [1]. The standard of care for patients with International Federation of Gynecology & Obstetrics (FIGO) Stage IB to IVA cervical cancer is definitive concurrent chemoradiation [2–6]. In cases of locally advanced cervical cancer, image-guided adaptive brachytherapy, including interstitial brachytherapy (ISBT), allows for improved dose conformity, dose escalation, and local control for these patients [7–9]. While a complete metabolic response (CMR) to treatment, as determined by positron-emission tomography integrated with computed tomography (PET-CT), predicts improved outcomes [10–12], 10–30% of cervical cancer patients do not achieve a CMR [4, 13]. The early identification of patients at risk of not achieving a CMR, who may benefit from local treatment escalation, could potentially improve care for this subset of patients.

Diffusion-weighted magnetic resonance imaging (DWI) has been explored in recent years as a noninvasive imaging technique providing insight into tumors’ cellular microenvironment by measuring the mobility of water molecules within tissue [14–16]. Apparent diffusion coefficient (ADC) values calculated from DWI offer a quantitative assessment of tumor cellularity as well as necrosis following therapy [17]. In general, water diffuses less easily in tissues with higher cellularity due to hindrances from cell membranes; it has been shown that malignant tumor tends to have lower ADC values [15], thought to be due to their denser cellularity, and conversely that tumor necrosis is associated with increases in ADC values [18]. Studies have shown that ADC values can predict malignancy and clinically relevant outcomes across multiple types of cancer [14, 16, 19, 20]. Recently, the prognostic value of ADC has been explored in cervical cancer specifically [21–25]. One study examined ADC measures at multiple percentiles (10th, 20th, etc.) and found that only 90th percentile ADC value was significantly associated with recurrence and survival in cervical cancer patients [25].

This study uniquely assessed the subset of patients with bulky cervical cancer, all of whom required ISBT. In this retrospective study, we sought to examine whether ADC values from post-external beam radiation therapy (EBRT), pre-ISBT clinical DWI could be used to predict complete response in women with bulky cervical cancer with a focus on the previously established 90th percentile cut point.

## Methods

### Patient selection

In this institutional review board-approved study, eligible patients were identified using our institution’s cancer registry database. All patients received concurrent chemoradiation with curative intent with ISBT for bulky cervical cancer (defined as High-Risk Clinical Target Volume (HR-CTV) > 30 cc). In this study, all patients received intensity-modulated radiation therapy (IMRT) to a microscopic dose of at least 4500 cGy to the pelvis in 25 fractions with simultaneous integrated boost and/or sequential boost to radiographically involved nodes. ISBT boost followed EBRT directly in 4 fractions delivered over 3 days. As our patients are treated in a closed, safety-net system, it is rare for patients not to complete treatment within the historic 56-day window.

All patients were treated at our institution between 2014 and 2019 and underwent PET-CT imaging at 3–6 months after brachytherapy to determine treatment response. Patients were excluded if pretreatment imaging was done at another hospital, if it did not include the necessary MRI sequences, or if it was of unacceptable quality; patients were also excluded if CMR could not be confirmed due to insufficient follow-up. Electronic health records (EHRs) were used to obtain demographic, disease, treatment, and imaging information. Patients were staged in accordance with 2018 FIGO staging classifications.

### Magnetic resonance imaging

MRIs were required for all patients before ISBT for planning purposes, in accordance with institutional protocol. Both structural and diffusion MRI images were acquired on either 1.5 T (GE Discovery MR450; *n* = 24) or 3 T scanner (GE Discovery MR750; *n* = 21).

Axial trace-weighted diffusion MRI images [26] were acquired with a twice-refocused spin-echo echo planner imaging (EPI) sequence [27] in two separate acquisitions with either low b value (bval = 500 s/mm^2^, or b500) or high b value (bval = 1000 s/mm^2^, or b1000) protocols: field of view (FOV) = 30 cm x 30 cm, matrix (readout x phase encoding) = 70 × 100 (1.5 T) or 100 × 150 (3 T), in-plane resolution = 4.3 mm x 3 mm (1.5 T) or 3 mm x 2 mm (3 T), slice thickness = 5 mm with 1 mm gap, echo spacing 0.40 ms (1.5 T) or 0.47 ms (3 T), echo time (TE) = 47 ms (1.5 T) or 49 ms (3 T) for b500 and 55 ms (1.5 T) or 58 ms (3 T) for b1000, repetition time (TR) ~ 3–4 s (1.5 T) or 4–5 s (3T), number of averages (NEX) = 8 (b500) or 10 (b1000). Apparent diffusion coefficient map was calculated with b = 0 images in each acquisition using vendor provided software on the scanner.

Axial T2-weighted (T2w) images were acquired with a fast spin echo sequence: FOV = 20 cm x 20 cm (1.5 T) or 18 cm x 18 cm (3 T), in-plane resolution = 0.8 mm x 0.8 mm (1.5 T) or 0.6 mm x 0.6 mm (3 T), slice thickness = 3.5 mm (1.5 T ) or 3 mm (3 T) with 1 mm gap, in-plane acceleration (PROPELLER) = 2, TE = 100.0 ms (1.5 T) or 102.3 ms (3 T), TR = 8.0 s (1.5 T) or 6.9 s (3 T), flip angle = 160° (1.5 T) or 120° (3T), bandwidth = 246 Hz/Pixel, echo spacing = 6.7 ms (1.5 T) or 7.3 ms (3 T).

### Image analysis

**Fig. 1:**
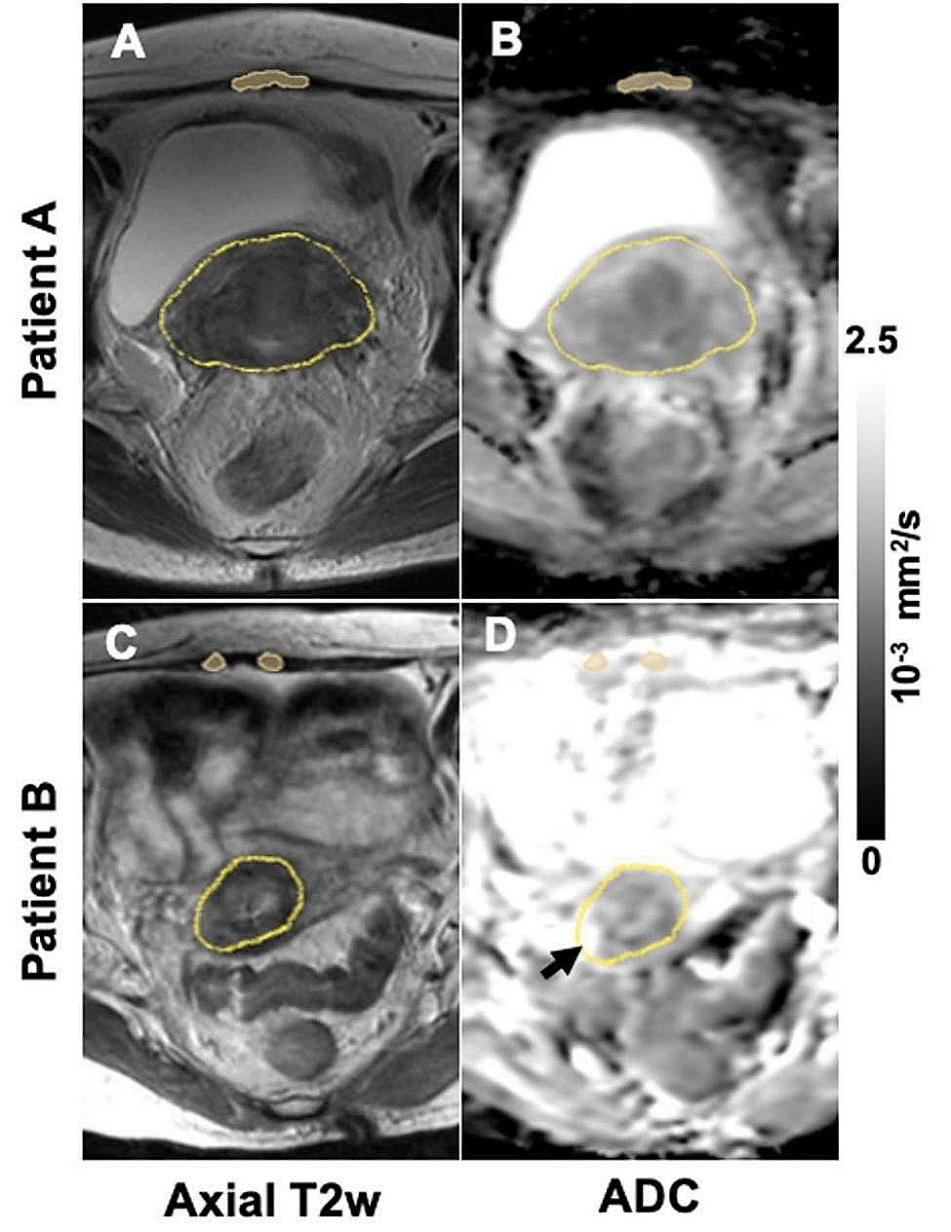
Representative axial T2w images (left column) and ADC maps (right column, from b500 protocol) of patient without (Patient **A**, upper row) and with (Patient **B**, bottom row) complete metabolic response. Contours of primary tumor are shown in the middle of the images. Bright area within the tumor contour of Patient B ADC map (black arrow) represents tumor necrosis, which contributes most to 90th percentile ADC. Contours of reference muscle tissue are also shown on the top of the images

Primary tumor/cervix and reference tissue segmentation was performed (by EED and reviewed by ML) on axial T2w images, typically spanning 3–7 slices, with 3D Slicer (Federov, et al. [28]).. In all cases, the entire cervix was contoured in addition to gross tumor, regardless of how well the primary tumor could be readily identified due to post-EBRT change. As such, segmentation of the tumor was analogous to the contouring of HR-CTV for brachytherapy. A separate contour of the rectus abdominis muscle was segmented for each patient as a reference tissue for ADC measurement. Muscle segmentations which were contaminated by partial volume effect were excluded. The axial T2w images were linearly registered onto the b = 0 image from the DWI using vendor-provided image analysis workstation. Subsequently, regions of interest (ROIs) were propagated onto the ADC maps for each patient (representative cases shown in Fig. 1). The mean and 90th percentile ADC value for all voxels included in the tumor ROIs were calculated.

### DWI quality assurance

**Fig. 2:**
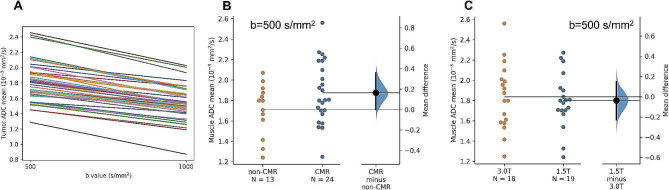
**(A)** parallel plot of tumor mean ADC from b500 and b1000 dMRI protocols in the same subject, **(B)** comparison of reference muscle tissue mean ADC (b500) between CMR and non-CMR cohorts, and **(C)** comparison of reference muscle tissue mean ADC (b500) between 3T and 1.5T cohorts

The quality of the DWI analysis was assessed by (i) checking for the expected pattern [29] of consistently lower tumor ADC values in b1000 than b500 DWI protocol from the same subject (Fig. 2A), (ii) comparing reference muscle tissue mean ADC in the b500 DWI protocol between CMR and non-CMR cohorts to verify a lack of significant ADC measurement bias (Fig. 2B), and (iii) comparing reference muscle tissue mean ADC in the b500 DWI protocol between data acquired at different (3T vs. 1.5T) field strengths to verify a lack of significant ADC measurement bias (Fig. 2C).

### Statistical analysis

The endpoint assessed was CMR, as recorded in the EHR. Decisions about whether a patient achieved CMR were based on PET-CT imaging 3–6 months after brachytherapy and were made by the patient’s attending radiation oncologist. Shapiro-Wilk test was used to assess normal distribution of ADC values. Given the known relationship of tumor malignancy and ADC, a one-sided pooled t-test was used to assess the significance of 90th percentile ADC values, with a *p-*value of 0.05 or less considered statistically significant. Youden’s J statistic was used to determine a 90th percentile ADC threshold that meaningfully distinguished between patients who achieved CMR and those that did not. Logistic regression analysis was used to assess for significant associations between CMR and multiple demographic, tumor, and treatment factors. A *p*-value of 0.1 or less was considered borderline significant in this univariate analysis and that factor was included in multivariate analysis. A *p-*value of 0.05 or less was considered statistically significant in the multivariate analysis. Odds ratios and confidence intervals were calculated to assess the effect of factors on CMR in univariate and multivariate analyses. Data analysis was performed in JMP 16.0 statistical software.

## Results

### Demographic, tumor, and treatment characteristics

Baseline demographic, tumor, and treatment characteristics are listed in Table 1. 53 patients met initial eligibility criteria. Of these, 8 were excluded for the following reasons: pretreatment imaging done at another hospital (*n* = 3), pretreatment imaging that did not include the necessary MRI sequences (*n* = 3), pretreatment imaging of unacceptable quality (*n* = 1), and lack of follow-up to confirm whether the patient achieved a CMR (*n* = 1). This left a total of 45 patients with usable data for this study. Forty patients (or 89%) had squamous cell carcinoma, and the remaining 5 (11%) had adenocarcinoma (*n* = 4) or adenosquamous (*n* = 1) carcinoma. The distribution of FIGO stages at diagnosis was as follows: 13 patients (29%) with Stage II, 22 patients (49%) with Stage III, and 10 patients (22%) with Stage IV cervical cancer. The median HR-CTV was 76.2 cc (range, 30.4–356.3 cc). The median EQD2 dose to HR-CTV was 85.0 Gy (range, 77.9–93.5 Gy). The median number of chemotherapy cycles received was 6 (range, 2–6). The median 90th percentile ADC of the primary tumor on pre-ISBT b500 DWI was 2.35 × 10^− 3^ mm^2^/s (range, 1.79–3.56 × 10^− 3^ mm^2^/s). The total number of cases with useful reference tissue segmentation was *n* = 37 (*n* = 18 3T and *n* = 19 1.5T). Twenty-eight patients (62%) achieved a CMR on follow-up PET-CT; 17 patients (38%) did not.

**Table 1:** Patient, tumor, and treatment characteristics

Characteristic	No. of Pts (%) (*n* = 45)
Median age at diagnosis, years (range)	47 (29–82)
Tumor Histology, *n* (%)	
Squamous Cell Carcinoma	40 (89%)
Adenocarcinoma/ Adenosquamous	5 (11%)
FIGO Stage, *n* (%)	
I	0
II	13 (29%)
III	22 (49%)
IV	10 (22%)
Chemotherapy cycles, median (range)	6 (2–6)
HR-CTV size in cc, median (range)	76.2 (30.4- 356.3)
90th percentile ADC, median (range)	2.35 (1.79–3.56)
Complete Metabolic Response	
Yes	28 (62%)
No	17 (38%)

*Abbreviations: FIGO = International Federation of Gynecology and Obstetrics; HR-CTV = high risk clinical target volume; ADC = apparent diffusion coefficient*

### Diffusion MRI

**Fig. 3:**
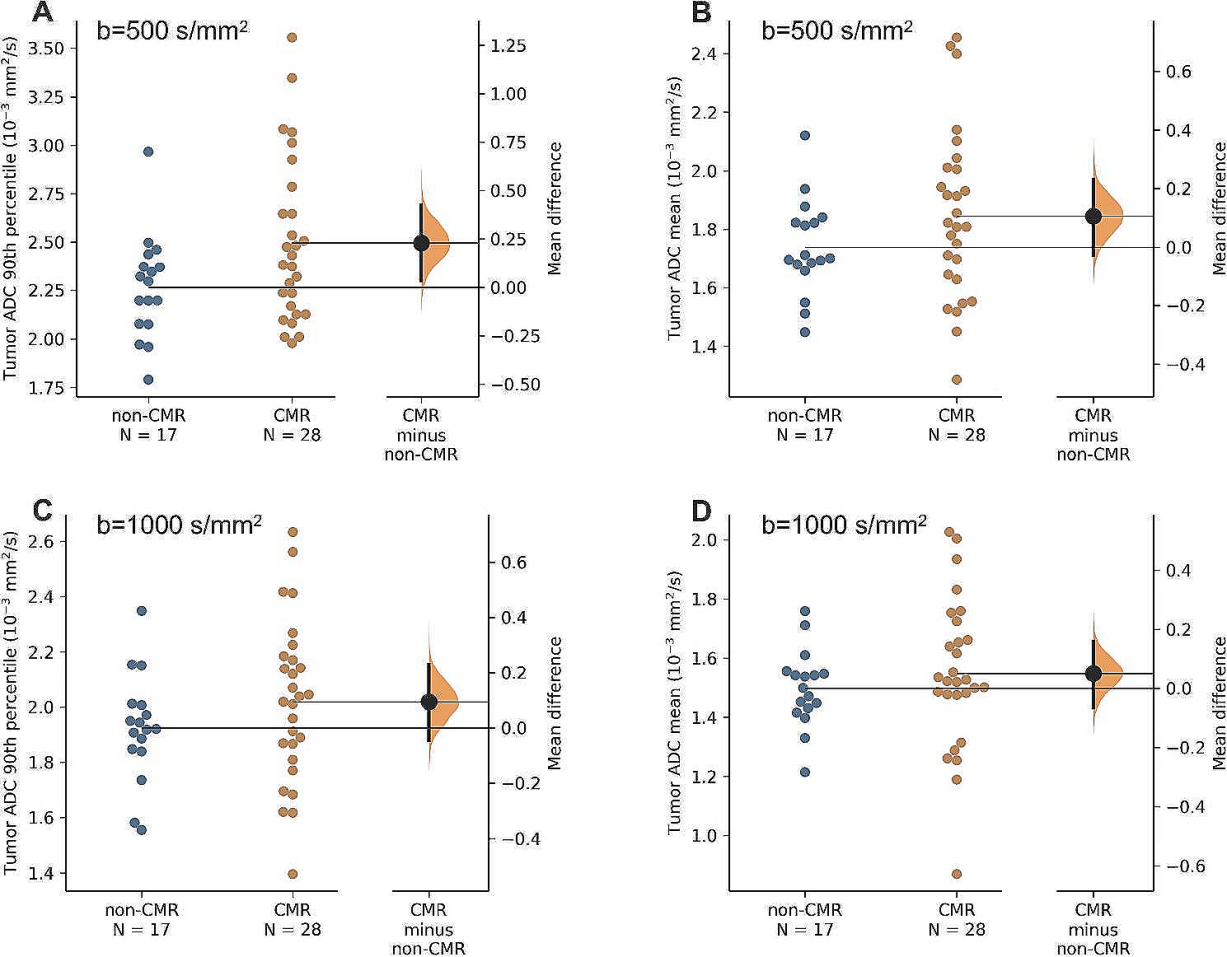
Comparisons of tumor 90th percentile ADC (**A** and **C**) and mean ADC (**B** and **D**) values between Complete Metabolic Response (CMR, *n* = 17) and non-CMR (*n* = 28) cohorts from b500 and b1000 dMRI protocols. Permutation-based mean difference (CMR minus non-CMR) estimation, as well as its posterior distribution (orange shaded area) and confidence interval (black bar), is plotted on the right of each figure. Among the comparisons, only tumor 90th percentile ADC from the b500 dMRI protocol **(A)** show a substantial effect size in the mean difference between the two cohorts

Between CMR and non-CMR cohorts, there was no significant difference in tumor mean ADC (b500, *p* = 0.18, Fig. 3B or b1000, *p* = 0.46, Fig. 3D). There was no significant difference in 90th percentile from the b1000 DWI protocol (*p* = 0.25, Fig. 3C). However, the 90th percentile ADC values from the b500 DWI protocol in the CMR group were substantially larger (mean difference = 0.23 × 10^− 3^ mm^2^/s, *p* = 0.048, Fig. 3A) than that in the non-CMR group, which hence is the focus of further analysis in this study.

A one-sided pooled t-test found that 90th percentile ADC from b500 DWI protocol was significantly associated with complete metabolic response (*p =* 0.0148). A closer inspection of Fig. 3A reveals that the group difference is mostly driven by cases with high 90th percentile ADC values in the CMR group (i.e., a substantial upward spread of ADC values in Fig. 3A that is not apparent in Fig. 3B-D) despite many overlapping cases with 90th percentile ADC values below ~ 2.5 × 10^− 3^ mm^2^/s. A 90th percentile ADC threshold of 2.10 × 10^− 3^ mm^2^/s was found from the b500 DWI protocol utilizing Youden’s Index, suggesting that patients above this threshold are 5.33 (95% CI, 1.35–24.4) times more likely to achieve CMR than those below.

Per the quality control assessment of our DWI analysis described above, we verified (i) the expected pattern [29] of lower tumor mean ADC values in b1000 than b500 DWI protocols (Fig. 2A), as well as the lack of significant ADC measurement bias using the reference muscle tissue mean ADC (ii) in the b500 DWI protocol between CMR and non-CMR cohorts (Fig. 2B) and (iii) in the b500 DWI protocol between data acquired at different (3T vs. 1.5T) field strengths (Fig. 2C).

### Factors associated with complete metabolic response (CMR)

Univariate and multivariate analyses results are in Table 2. In the univariate analysis for CMR, only 90th percentile ADC values from b500 DWI protocol were significant (*p* = 0.029). There were no significant associations found on univariate analysis between CMR and FIGO stage, histology, tumor size, HR-CTV BED (Gy_10_) which is directly related to the EQD2 prescribed dose, pre-treatment hemoglobin, or patient age. Borderline significant factors were ISBT HR-CTV size in cc (*p =* 0.054), and number of chemotherapy cycles (*p =* 0.078). When 90th percentile ADC, ISBT HR-CTV size, and number of chemotherapy cycles were included in a multivariate analysis with adjustment for false-discovery rate (i.e., multiple comparisons), only 90th percentile ADC (*p <* 0.0001) and ISBT HR-CTV (*p <* 0.003) were significant.

**Table 2:** Univariate/multivariate analysis of factors associated with complete metabolic response

	Univariate			Multivariate		
	Unit Odds Ratio	95% CI	*p*-value	Unit Odds Ratio	95% CI	FDR *p*-value
90th percentile ADC (10^− 3^ mm2/s)	1.002	1.0004–1.004	**0.029**	1.004	1.001–1.007	**< 0.0001**
ISBT HR-CTV (cc)	0.982	0.962–0.997	**0.054**	0.979	0.942–0.998	**< 0.003**
Number chemotherapy cycles (cycle)	1.806	0.970–3.742	**0.078**	2.330	0.979–7.889	0.057
FIGO stage	-	-	0.14	-	-	-
HIV status	-	-	0.19	-	-	-
Smoking status	-	-	0.22	-	-	-
Tumor size	-	-	0.22	-	-	-
HR-CTV BED (Gy_10_)	-	-	0.63	-	-	-
Histology	-	-	0.65	-	-	-
Pre-treatment hemoglobin	-	-	0.68	-	-	-
Age at treatment	-	-	0.81	-	-	-

*Abbreviations: HR-CTV = High-Risk Clinical Target Volume, FIGO = International Federation of Gynecology and Obstetrics*

*BED = Biologically Effective Dose, FDR = False-Discovery Rate*

## Discussion

Our results suggest that DWI, and specifically 90th percentile ADC value, has clinical relevance in risk-stratifying patients undergoing ISBT for bulky cervical cancer. We also explored the potential for identifying a 90th percentile ADC threshold that can differentiate patients based on likelihood of complete response to brachytherapy.

While multiple studies in recent years have explored the utility of ADC values in predicting outcomes for cervical cancer in general [24, 25, 30–32], little attention has been given to the subset of patients who require ISBT after EBRT. These patients represent a high-risk population and thus would greatly benefit from improved prognostication. Our work suggests that the prognostic utility of ADC values for cervical cancer treated with EBRT can be extended to those tumors requiring ISBT.

Despite mixed results as to whether lower ADC values portend a better or worse prognosis [23, 33], the literature in recent years has generally supported an association between higher ADC values and better outcomes [24, 25, 31, 32, 34–36]. Our results are in line with this finding. Figure 3A illustrates an important bimodal distribution: while lower 90th percentile ADC values are spread between the CMR and non-CMR groups, the cohort of patients with high 90th percentile ADC values ( ≥ ~ 2.5 × 10^− 3^ mm^2^/s) almost universally achieved a CMR. The upward spread of ADC values in these DWI images likely represents tumor necrosis after EBRT (e.g., Fig. 1D, Patient B, black arrow in the ADC map), portending a favorable response to ISBT [34].

Despite consensus on the direction of this association, agreement has not yet been reached as to ADC cutoff values that could be used to risk-stratify cervical cancer patients. Ho, et al. found a 90th percentile pretreatment ADC cutoff value of 1.917 × 10^− 3^ mm^2^/s, with lower values associated with worsened outcomes [25], while our data suggests a 90th percentile ADC cutoff value of 2.10 × 10^− 3^ mm^2^/s. Two notable distinctions are that in our analysis our scans were all acquired following EBRT and prior to BT (rather than at baseline) and our focus was on metabolic response, given our population of bulky local disease, rather than survival or progression-free survival. Ultimately, patients in our cohort with a 90th percentile ADC value above this threshold were 5.3 times more likely to achieve a CMR.

Importantly, our DWI analysis reaffirms that the relevance of percentile ADC threshold is b value dependent. For our study, 90th percentile ADC threshold from b500 distinguishes a subgroup of patients between the CMR and non-CMR group, while 90th percentile ADC from b1000 obscures such distinction in the high ADC regime. This is because of the noise floor effect of diffusion weighted image signal in the tumor necrosis region [37], which contains almost freely diffusing tissue water (hence high b value diffusion weighting crushes the signal down to noise floor) and contributes most to the 90th percentile ADC. Choosing an optimal b value for similar studies hence depends on ensuring sufficient signal-to-noise ratio for the tumor necrosis region. As it is well-known that ADC value depends on many technical details (e.g., b value) of the diffusion MRI protocol, the threshold found in this study has limited generalizability beyond a single site or across MRI scanner vendors. Further studies drawn from multiple institutions are needed to explore appropriate ADC cutoffs and their role in clinical practice.

Additionally, manual delineation of the tumor region and whole cervix is subject to error, especially at uncertain boundaries. Transformation of regions drawn on T2w anatomical images to ADC maps further compounds any boundary errors. Thus, ADC percentile measures are inevitably sensitive to error in ROI boundary definition and may be influenced by expertise in tumor identification; the impact of this represents another area for future study.

Lastly, the small cohort size of this study limited the strength of some of our statistical measures, hence the exploratory nature of our univariate and multivariate analyses of the potential of DWI in clinical prognostication. Further, while data support that complete metabolic response on PET-CT following therapy is associated with improved overall survival and a reduced risk of isolated local recurrence [10–12], we acknowledge that it is remains a surrogate endpoint for actual local failure or disease-free survival. In addition, our diffusion MRI protocols contain imaging parameter variations (e.g., field strength, TR, number of slices, etc.) which is not uncommon in clinical practice (as compared to a prospective research study). Despite this limitation, the quality assurance of our DWI analysis (Fig. 3) rules out common systematic measurement biases in our ADC measurement and adds confidence in designing larger studies in real-world clinical radiology sites.

## Conclusions

Our work supports existing literature in suggesting a role for 90th percentile ADC values from DWI to predict cervical cancer outcomes, while extending this line of inquiry to patients with bulky disease, many of whom require interstitial brachytherapy and may still not achieve a complete response to treatment. Additionally, our data further explores an optimal 90th percentile ADC value threshold that may serve to risk-stratify future patients. The identification of patients at risk of nonresponse to ISBT could allow for better tailoring of treatment and improved outcomes for this high-risk patient population.

## Data Availability

All data generated and analyzed during this study are available from the corresponding author on request.

## Abbreviations

ADC: Apparent diffusion coefficient
BED_10_: Biologically Effective Dose at alpha beta of ten
CMR: Complete metabolic response
DWI: Diffusion-weighted magnetic resonance imaging
EBRT: External beam radiation therapy
EHR: Electronic health record
EQD2: Normalized treatment dose in conventional fractionation
FIGO: International Federation of Gynecology & Obstetrics
HR-CTV: High-Risk Clinical Target Volume
ISBT: Interstitial brachytherapy
PET-CT: Positron-emission tomography integrated with computed tomography
ROI: Region of interest
